# Thigh musculature stiffness during active muscle contraction after anterior cruciate ligament injury

**DOI:** 10.1186/s12891-020-03342-x

**Published:** 2020-05-21

**Authors:** April L. McPherson, Nathaniel A. Bates, Clifton R. Haider, Takashi Nagai, Timothy E. Hewett, Nathan D. Schilaty

**Affiliations:** 1 Mayo Clinic Graduate School of Biomedical Sciences, Mayo Clinic, Rochester, MN USA; 2 Department of Orthopedic Surgery, Mayo Clinic, 200 First St SW, Rochester, MN 55905 USA; 3 Department of Physiology & Biomedical Engineering, Mayo Clinic, Rochester, MN USA; 4 Sports Medicine Center, Mayo Clinic, Rochester, MN USA; 5 Special Purpose Processor Development Group, Department of Physiology and Biomedical Engineering, Mayo Clinic, Rochester, MN USA; 6 Sparta Science, Menlo Park, CA USA; 7 Department of Physical Medicine & Rehabilitation, Mayo Clinic, Rochester, MN USA

**Keywords:** Shear wave elastography, EMG decomposition, Rehabilitation, ACL reconstruction, Arthrogenic muscle inhibition

## Abstract

**Background:**

Altered motor unit (MU) activity has been identified after anterior cruciate ligament (ACL) injury, but its effect on muscle tissue properties is unknown. The purpose of this study was to compare thigh musculature muscle stiffness between control and ACL-injured subjects.

**Methods:**

Thirty ACL-injured subjects and 25 control subjects were recruited. Subjects completed a randomized protocol of isometric contractions while electromyography (EMG) signals were recorded. Three maximum voluntary isometric contractions (MVIC) determined peak force for 10 and 25% MVIC trials. Shear wave elastography was captured during each 10 and 25% MVIC trials.

**Results:**

Differences in muscle stiffness were assessed between limbs and groups. 12 months post-surgery had higher stiffness for VM 0% MVIC, VL 0 and 10% MVIC, and ST 10 and 25% MVIC (all *p* ≤ 0.04).

**Conclusion:**

Thigh musculature stiffness changed throughout rehabilitation and remained altered at 12 months after ACL reconstruction.

## Background

Quadriceps atrophy, strength, and activation deficits persist after anterior cruciate ligament (ACL) injury and reconstruction (ACLR) [1–3]. Moreover, neuromuscular adaptations after ACL injury may provide compensatory mechanisms to overcome loss of neurosensory information from the native ACL [4, 5]. Recent work on motor unit (MU) rate coding revealed that individuals at 12 months post-ACLR surgery exhibited lower rate coding (the rate at which a MU generates action potentials) of quadriceps MU concomitant to higher rate coding of hamstring MU compared to controls [6]. Similarly, lower quadriceps neuromuscular activation, determined by the median frequency of the electromyography (EMG) recordings, was reported in the ACL-injured limb than the non-injured limb as well as lower torque generation at approximately 7.5 months post-ACLR surgery [4]. Results of these studies indicate that MU activity may not normalize relative to control subjects by 12 months post-surgery. This is consistent with other biomechanical and clinical reports of unresolved deficits at 12 months post-surgery [5, 7].

Neural activity controls muscle activation, contraction, and force output [8]. However, contributions of neural activity to mechanical properties of muscle (e.g. muscle tissue stiffness) in individuals with ACL injury and ACLR have not been extensively investigated. Involved limb in individuals with ACL injury had higher hamstring stiffness (as modeled by a mass spring system), and higher stiffness values were positively correlated to functional outcomes [9, 10]. Contrarily, leg stiffness as measured by force plate assessments was observed in the involved limb in ACLR individuals during a unilateral hopping protocol [4]. Alternatively, shear wave elastography (SWE) provides a reliable and quantitative assessment of muscle stiffness and has not been investigated after ACL injury and ACLR [11–14]. Shear wave elastography provides a quick and noninvasive measurement of muscle stiffness, estimated from measurement of muscle shear modulus or the tissue’s resistance to shear deformation, and is an important factor for physical function, movement, and athletic performance. Shear modulus, a surrogate estimate for stiffness, increased linearly with greater contraction level and greater EMG amplitude in the biceps brachii and tibialis anterior in healthy subjects [15, 16]. However, this relationship has not been reported after ACL injury.

Therefore, the objective of the current study was to compare thigh musculature stiffness between control and ACL-injured subjects. It was hypothesized that stiffness of the ACL-injured limb would be decreased compared to the non-injured limb and compared to healthy controls. Second, it was hypothesized that stiffness would increase linearly with contraction level, but the relationship would vary between control and ACL-injured subjects. Finally, an exploratory analysis between MU rate coding and stiffness was performed; it was hypothesized that as MU rate coding increased, muscle stiffness would increase linearly.

## Methods

**Fig. 1:**
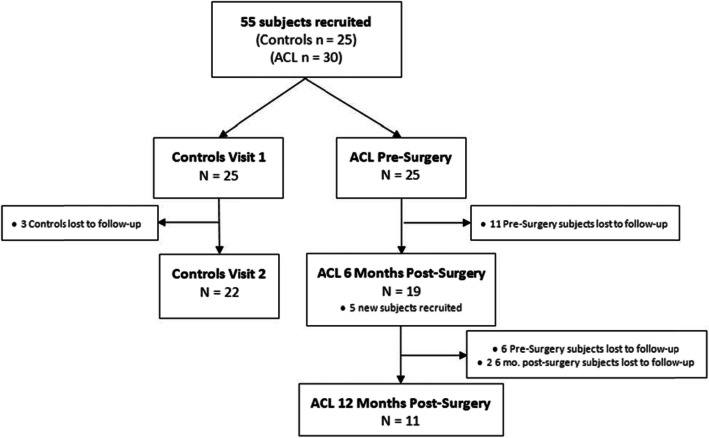
Subject recruitment and follow-up

The Mayo Clinic Institutional Review Board approved the study (16–010600). Twenty-five subjects with no history of previous ACL injury were recruited (Fig. 1**,** Table 1). Thirty subjects with an ACL injury were recruited at one of two time points: pre-ACL reconstruction (ACLR) or 6 months post-ACLR (6 months, ± 1 month). ACL-patients were included if they were between 14 and 25 years old with a confirmed ACL injury diagnosis and subsequent ACL reconstruction by an orthopedic surgeon. Pre-surgery patients were tested, on average, 55 days (SD 66, range 10–320 days) after the date of ACL injury. Subjects were followed longitudinally for testing intervals of 6 months (± 1 month). 76% of ACLR subjects had a bone-patellar-tendon bone autograft, 21% of subjects had a semitendinosus autograft, and 3% had an allograft. Criteria for inclusion were healthy active individuals 14 to 25 years old recreationally active a minimum of 3 days per week. Exclusion criteria were: lower extremity injury (other than ACL) in the previous 6 months, neurological disorders, paralysis, neuromuscular disease, cardiovascular disease, exercise-induced injury (other than ACL), asthma, and pregnancy. Control subjects’ non-dominant leg was compared to the ACL-injured limb to eliminate the potential bias introduced by comparing a control subject’s dominant to an injured limb. Subjects self-reported their dominant limb, defined as the preferred kicking leg.

**Table 1:** Population Demographics

	**Variable**	**Age (yrs)**	**Height (cm)**	**Mass (kg)**
Group	Control (*n* = 25)	18.8 (3.1)	173.9 (8.5)	67.6 (13.4)
	ACL Injured (*n* = 30)	19.1 (3.2)	173.6 (9.0)	77.3 (15.6)^b^
Timepoint	Pre-Operative (n = 25)	18.8 (3.0)	173.5 (9.2)	75.9 (14.0)
	6-month (*n* = 19)	19.3 (3.1)	172.3 (11.1)	77.2 (16.7)
	12-month (*n* = 11)	19.8 (3.2)	173.2 (10.7)	73.1 (16.6)

Divided by Group and by Time Point. Values are expressed as mean (SD).

^b^Indicates significant difference between groups (*P* < 0.05)

Prior to testing, subjects completed the following International Knee Documentation Committee questionnaire, the ACL-Return to Sport after Injury scale, and Marx Activity Rating Scale. Testing was performed on a HumacNORM (CSMi, Stoughton, MA). A custom load cell apparatus (MLP-300; Transducer Techniques, Temecula, CA) affixed to the HumacNORM torque arm was used to measure the subject’s force effort required for the EMG decomposition software (EMGWorks (v4); Delsys, Natick, MA). For isometric knee extension testing, subjects were positioned in a seated position with their leg at 80° flexion (0° = full extension). Subjects were secured with straps at the shoulder and waist to minimize whole body movement. Surface EMG electrodes (Delsys, Natick, MA) were placed on the muscle belly of both the vastus medialis (VM) and vastus lateralis (VL) muscles according to SENIAM standards [17]. For isometric knee flexion testing, subjects lay prone on the HumacNORM chair with their leg positioned at 30° knee flexion (0° = full extension). Surface EMG electrodes were placed on the muscle belly of the biceps femoris (BF) and semitendinosus (ST) muscles. Skin was shaved and cleansed with an alcohol wipe prior to electrode placement to ensure quality skin-electrode contact.

A GE Logiq E9 Ultrasound System with SWE capabilities was used to assess muscle stiffness (GE Healthcare, Wauwatosa, WI) with a 9 L linear array probe (2–8 MHz). The ultrasound B-mode image was used to localize the target muscle tissue and a rectangular field of view was selected for SWE acquisition. The ultrasound transducer head was aligned parallel with the muscle fibers to obtain shear modulus values in the region of interest [11]. Probe placement was consistent between subjects – for the quadriceps musculature, the probe was placed immediately inferior to the EMG electrodes; for the hamstrings, the probe was placed immediately superior to the electrodes.

### Testing protocol

A randomized protocol was generated for each subject to test both legs. Limb side (right vs. left), muscle group (hamstring vs. quadriceps), and order of trials (0–25%) were each randomized. Three isometric knee flexion and extension maximum voluntary isometric contractions (MVIC) were performed to determine maximum strength (F_max_) and the 10, and 25% MVIC contraction levels for each leg. After the MVIC trials, the randomized protocol was followed. Each test consisted of following a trapezoidal waveform, with a sustained 10 second contraction during which three shear wave elastography images were obtained. The subject was instructed to follow the trapezoid, with real-time feedback of force production displayed on a computer monitor. Each trial was repeated twice in order to obtain SWE data on both muscles in the muscle group.

### EMG acquisition and decomposition

Four channels of analog data were collected at 20 kHz for the 5-pin sensor array on the EMG electrode. A high-pass filter with a 20 Hz cutoff frequency and a low pass filter with a 1750 Hz cutoff frequency were applied to the analog signal and the resultant digital signal was stored [18]. EMG decomposition was performed to obtain MU activity, defined as average MU rate coding per second (pps, pulses per second) [19, 20]. When decomposition was initiated, the digital signal was filtered with a high-pass 50 Hz cutoff frequency filter to remove the long tails of action potentials and reduce the incidence of superposition between action potentials [18]. The decomposition algorithm uses artificial intelligence framework to extract action potential templates of MU action potential trains from the EMG signal. The algorithm then searches for superposition using constructive and deconstructive interference effects [18]. MUs that demonstrated < 90% accuracy using the software’s decompose-synthesize-decompose-compare method were not included in analyses [21].

### Data analysis

Shear wave velocity measured from the region of interest on the shear wave elastogram can be used to calculate shear modulus and stiffness (kPa) [11]. Muscle stiffness values were calculated for each trial using custom MATLAB software. The average stiffness value for each image (*n* = 3) in a trial was averaged; this average stiffness value for each muscle for each trial was used for analysis. Control subjects’ stiffness was compared between visits. As there were no significant differences (*p* > 0.05), stiffness from the first control visit was used.

Descriptive statistics were calculated for each group (Control, Pre-Surgery, 6 mo, 12 mo) (JMP 14, SAS Institute Inc., Cary, NC). F_max_ was normalized to body mass (N*kg^− 1^) before statistical analysis. Wilcoxon Signed Rank tests were used to test for between limb differences in F_max_. Due to recruitment at two different time points after ACL injury, only eight subjects were tested at all three time points after ACL injury. In addition to SWE data loss (*n* = 3), this resulted in five subjects with complete data. Therefore, each ACL time point is treated as an independent group and is compared only to the control group. Wilcoxon Rank Sum tests were used to evaluate differences between the control group and each ACL group for both F_max_ and Marx Activity Level scores. Stiffness was compared between limbs for each %MVIC using a Wilcoxon Signed Rank test for each group. A Wilcoxon Rank Sums test was used to assess the difference between the control group and the ACL-injured limb for each trial. Pearson’s correlations were used to analyze the relationship between stiffness and MU average rate coding. MU rate coding was normalized to force for analysis. Pearson’s correlation coefficients were classified as: very high (0.90–1.00), high (0.70–0.90), moderate (0.50–0.70), low (0.30–0.50), and negligible (0.00–0.30) [22]. Significance was set a-priori at *p* ≤ 0.05.

## Results

Fifty-five subjects were recruited; 25 control subjects (male *n* = 11; 44%) and 30 ACLR subjects (male *n* = 13; 43%). Marx Activity Level was significantly different between 12 months post-ACLR and controls (*p* = 0.05, all other *p* ≥ 0.39; Table 2). Non-injured quadriceps F_max_ was greater than the injured limb for the pre-surgery and 6 month groups (*p <* 0.001). In all three ACL-injured groups, hamstrings F_max_ was significantly different between limbs (*p* ≤ 0.05); however, in the 12 month group the injured hamstring was stronger than the non-injured limb. There were no differences in quadriceps F_max_ between the control group and any ACL-injured group for either limb (*p* ≥ 0.08). Similarly, there were no differences in non-injured hamstring F_max_ between the control group and any ACL-injured group (*p* ≥ 0.07). Injured-limb hamstrings F_max_ was significantly different from controls for the 12 month group (*p =* 0.02).

**Table 2:** Marx Activity Level (points), knee extensor strength (N*kg^− 1^), and knee flexor strength (N*kg^− 1^)

**Variable**		**Control (*n*** **= 25)**	**Pre-Surgery (*n* = 25)**	**6 month (*n*** **= 19)**	**12 month (*n*** **= 11)**
Marx Activity Level		16.0 (5.0)	15.5 (2.8)	14.0 (10.0)	10.0 (10.0)^c^
Quadriceps F_max_, N*kg^−1^	Injured	7.1 (4.0)	6.9 (2.6)	5.6 (3.4)	7.9 (2.7)^c^
	Non-injured	7.5 (4.4)	8.4 (2.6)^b^	8.5 (5.1)^b^	9.2 (3.7)
Hamstrings F_max_, N*kg^−1^	Injured	3.9 (2.4)	4.1 (1.7)	4.2 (2.3)	5.0 (3.1)
	Non-injured	4.0 (1.9)	4.7 (1.3)^b^	4.6 (1.6)^b^	4.7 (2.0)^b^

Divided by Group. Values are expressed as median (IQR). N, Newton.

^b^Indicates significant difference between limbs (*P* < 0.05)

^c^Indicates significant difference between ACL-injured group and controls (*P* < 0.05)

### Quadriceps muscle stiffness

**Fig. 2:**
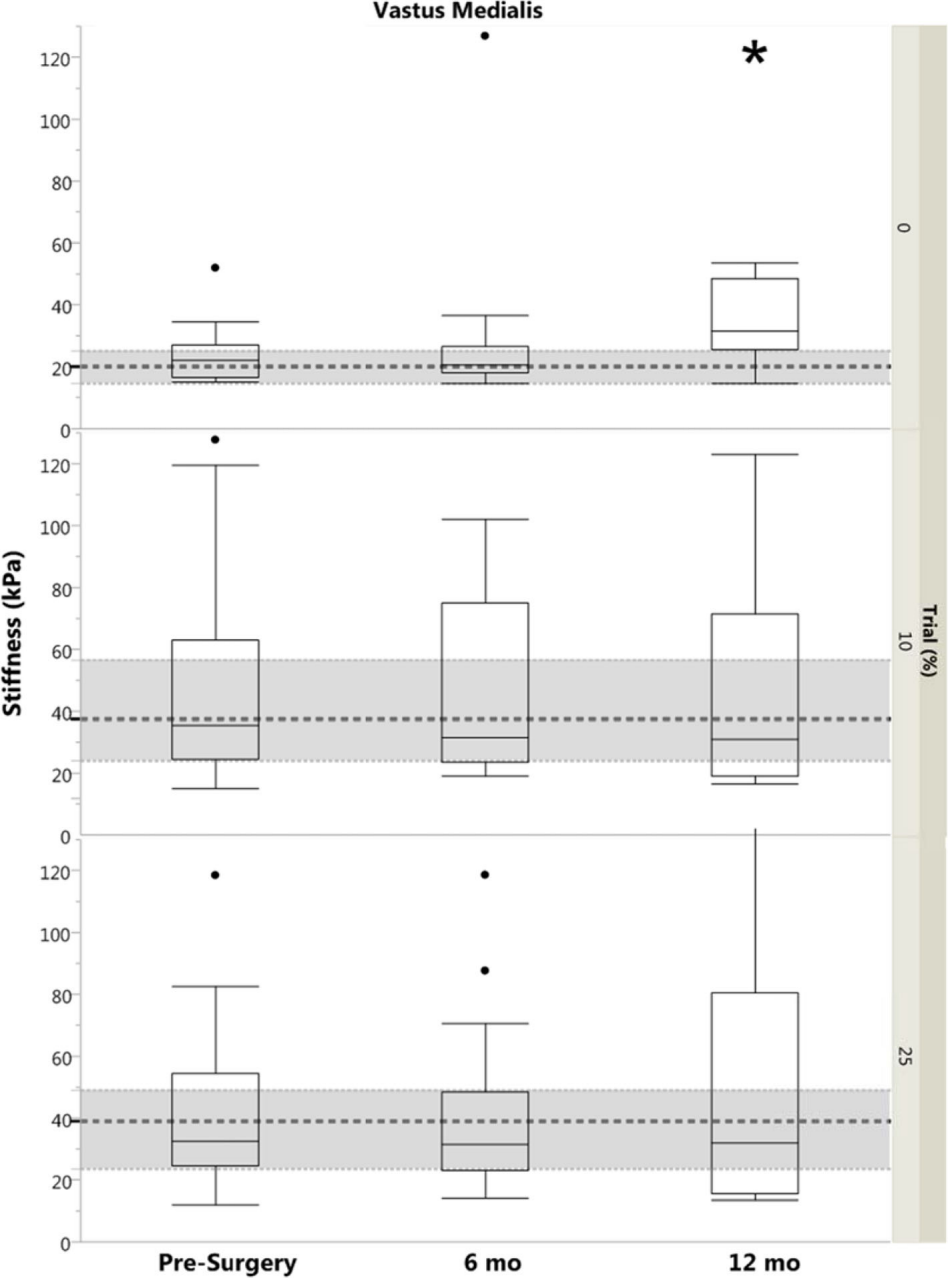
Box plot with outliers of Vastus Medialis Stiffness (by Group and by Trial). Dashed regions represent control group median and IQR. Significance designation (*) indicates difference between ACL-injured limb and control group (*p* < 0.05). kPa, kilopascal

**Fig. 3:**
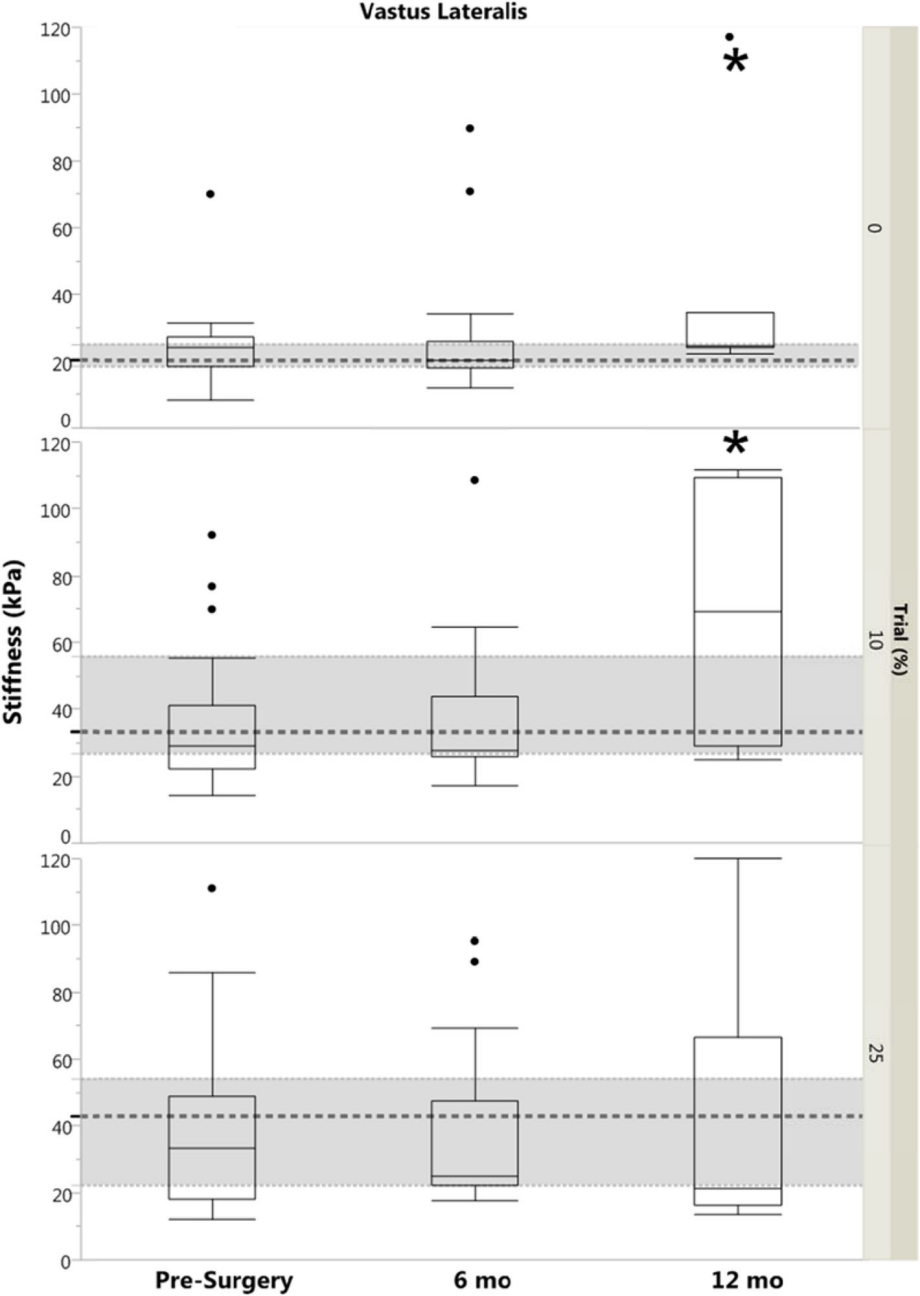
Box plot with outliers of Vastus Lateralis Stiffness (by Group and by Trial). Dashed regions represent control group median and IQR. Significance designation (*) indicates difference between ACL-injured limb and control group (*p* < 0.05). kPa, kilopascal

The only significant limb difference for VL stiffness was the 0% MVIC trial for the 6 month group, where non-injured limb stiffness was higher (*p* = 0.04). For the VM, there were significant differences between limbs for 25% MVIC in the 6 month group (*p* = 0.04) and for 0% MVIC for the 12 month group (*p* < 0.01), with higher stiffness in the injured limb compared to the non-injured limb. Injured limb stiffness was higher in the 12 month group compared to controls for VM 0% MVIC (*p* < 0.01; Fig. 2) and for VL 0 and 10% MVIC (*p* ≤ 0.04; Fig. 3).

### Hamstrings muscle stiffness

**Fig. 4:**
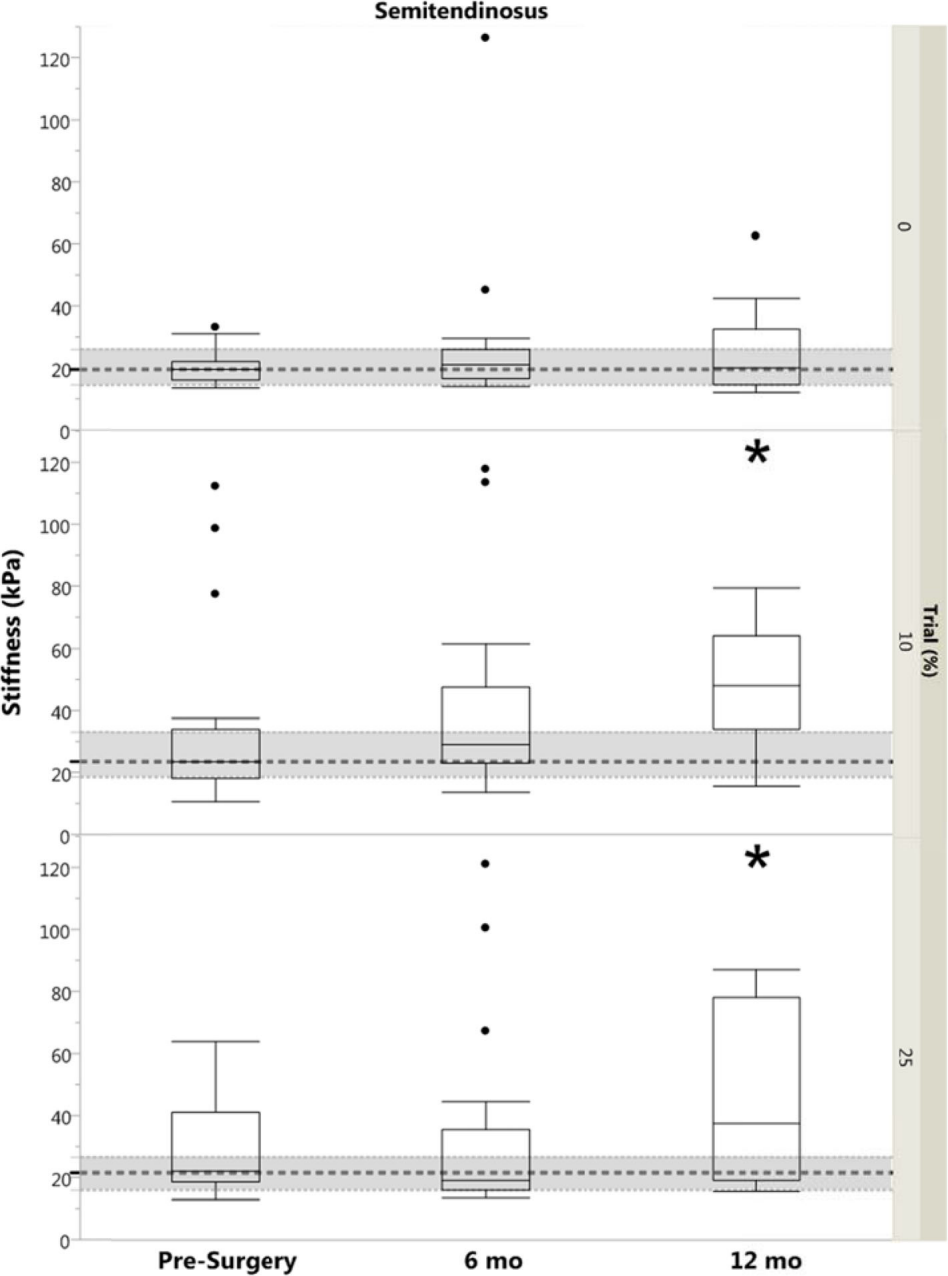
Box plot with outliers of Semitendinosus Stiffness (by Group and by Trial). Dashed regions represent control group median and IQR. Significance designation (*) indicates difference between ACL-injured limb and control group (*p* < 0.05). kPa, kilopascal

**Fig. 5:**
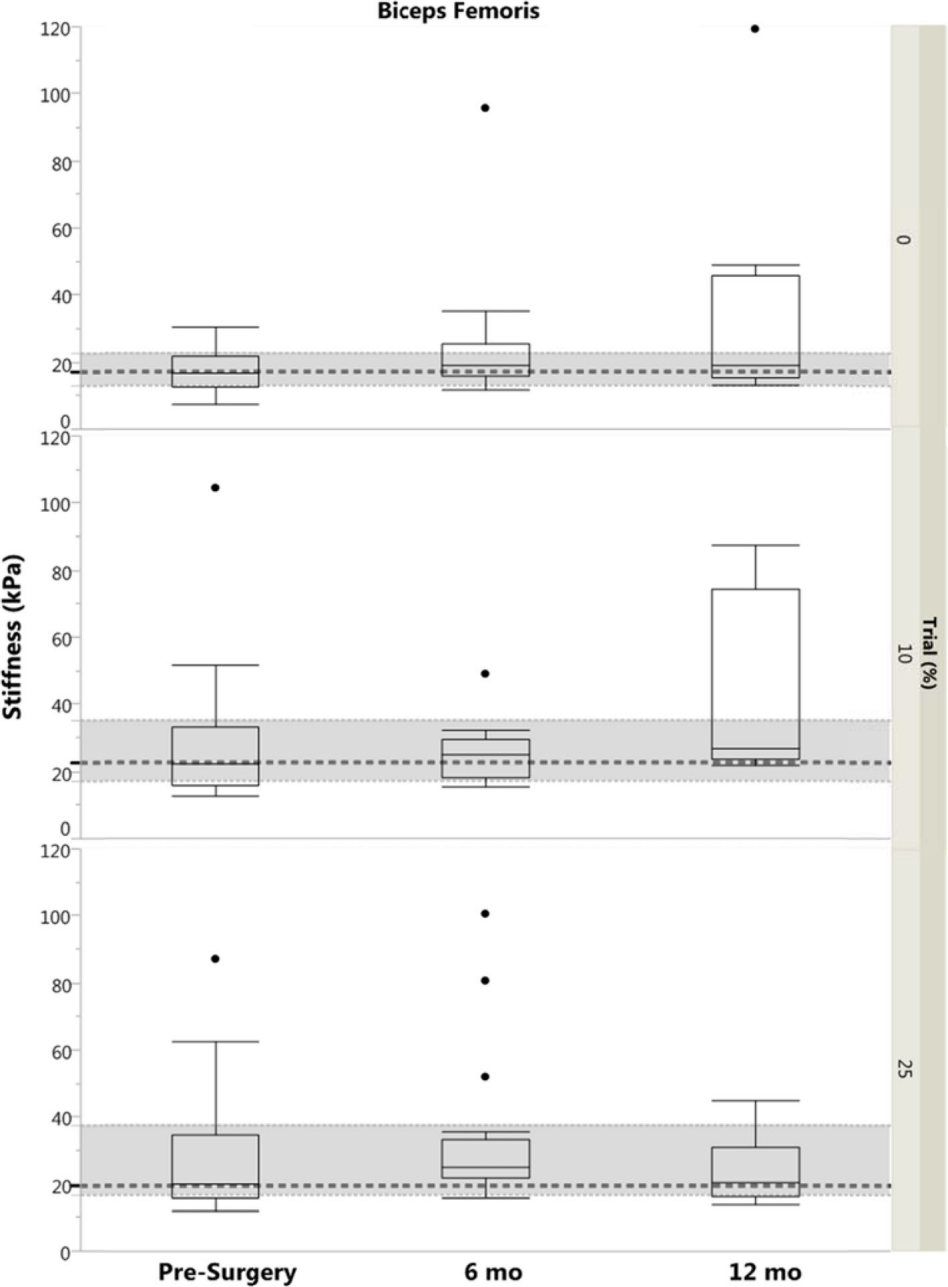
Box plot with outliers of Biceps Femoris Stiffness (by Group and by Trial). Dashed regions represent control group median and IQR. Significance designation (*) indicates difference between ACL-injured limb and control group (*p* < 0.05). kPa, kilopascal

There were no significant differences between limbs for either the BF or ST in any group or trial (*p* ≥ 0.13). Injured limb ST stiffness was higher in the 12 month group compared to controls for both 10 and 25% MVIC (*p* ≤ 0.04; Fig. 4). There were no other differences between controls and ACL-injured groups for either the BF or ST (*p* ≥ 0.07; Fig. 5).

### Motor unit rate coding and muscle stiffness

Linear regression analyses indicated a low relationship between MU average rate coding and stiffness for both 10 and 25% MVIC for the VM in the 6 month group (r ≥ 0.32). A low correlation was observed for the VL in the 25% MVIC trial control group and 10% MVIC group in the 12 month group. The only low correlations for the BF was 10% MVIC trial (r = 0.41) and for the ST was 25% MVIC (r = 0.47), both in the 6 month group.

## Discussion

The purpose of this study was to investigate how stiffness differs after ACL injury and compared to healthy control subjects. Identification of this relationship may help to delineate possible causes of impaired quadriceps strength after ACL injury. ACL-injured subjects in the current study indicated activity levels that were higher than previous literature for pre-surgery and 6 months post-surgery, but similar at 12 months, as measured by the Marx Activity Level scale (Pre-Surgery, 15.5 vs. 12 [23], 11 [24] points; 6 mo, 14 vs. 8 [25] points; 12 mo, 10 vs. 10 [24], 12 [26] points). Contrary to the hypothesis, there were minimal differences in stiffness between the injured and non-injured limbs. As hypothesized, there were differences in stiffness between controls and 12 months post-ACLR; however, the ACL-injured limb had higher stiffness than controls. The final exploratory hypothesis tested was not supported, as no distinct linear relationship was observed between MU rate coding and stiffness for any group.

Previous studies that examined the relationship between stiffness and %MVIC reported conflicting results, dependent on the range of %MVIC tested. For the full range of contraction intensity, shear modulus exhibited a linearly increasing relationship during fifth digit abductions [27]. However, this relationship becomes less distinct in larger muscle groups. Shear modulus appeared to follow a linearly increasing relationship in the biceps brachii, when static elbow flexion was tested at 15, 30, 45, and 60% MVIC [15]. However, an earlier study reported a curvilinear association up to 40% MVIC [28]. Results from the current study also show variability in the relationship between stiffness and %MVIC, group, and muscle. For example, the relationship appeared to linearly increase for the control group quadriceps whereas the 12 month group demonstrated slightly decreased VL stiffness as contraction intensity increased. However, SWE variability was greater in the 25% MVIC trials and thus the SWE data may be less reliable than the 0 and 10% MVIC trials and contribute to the indistinct relationship between contraction intensity and stiffness reported in the current study. Additional measurements at higher contraction levels may help to better delineate the true relationship between effort level and stiffness. While 35 and 50% MVIC trials were performed in the current study, technical limitations of the commercially-available ultrasound contributed to shear wave elastogram quality that was unfit for inclusion in the analyses. Previous work has also identified this technical limitation, in which higher stiffness with higher contraction levels in which the shear modulus was artefactually underestimated [28]. Further investigation is warranted with additional subjects to characterize the relationship between contraction level and stiffness with improved elastography methods that include a higher ceiling limit of kPa, real-time SWE data acquisition, or external vibration that does not rely on the push pulse [29].

A previous study of patients after knee joint surgery reported differences between resting-state and active contraction stiffness in the quadriceps muscles using a different ultrasound SWE device [30]. Moreover, the stiffness of the muscles in the affected limb was decreased relative to the non-affected limb during the active contraction [30]. In the current study, 0% VM stiffness was higher than controls in the 12 months group, but no differences were observed during active contraction. VL stiffness, however, was higher than controls in the 12 month group during both the 0 and 10% MVIC trials. Interestingly, quadriceps MU rate coding was significantly lower than controls for all trials at 12 months post-surgery in a previous study but recruitment threshold, or the force at which the MU first fired continuously, was higher than controls [6]. Similarly, ST stiffness was higher than controls in the 10 and 25% MVIC trials. Opposite of the quadriceps, at 12 months post-surgery, hamstrings MU rate coding and recruitment was higher than controls [6]. -injured subjects exhibited minimal differences in stiffness compared to controls in the current study, but significant differences in MU recruitment thresholds and rate coding [6]. Together, these findings may indicate that MU activity is not the sole contributor to stiffness during active muscle contraction. In addition, these findings may show that impaired quadriceps strength observed in ACL-injured subjects in clinical settings during rehabilitation may not be caused by altered stiffness.

There are several intrinsic and extrinsic factors that contribute to muscle stiffness that were not assessed in the current study. During an active muscle contraction stiffness increases, but the magnitude and relationship is dependent on the muscle and fascicle length, as well as the intensity and type of force [12]. Collagen content within the extracellular matrix is also a key contributor to increased passive stiffness [31, 32].

**Fig. 6:**
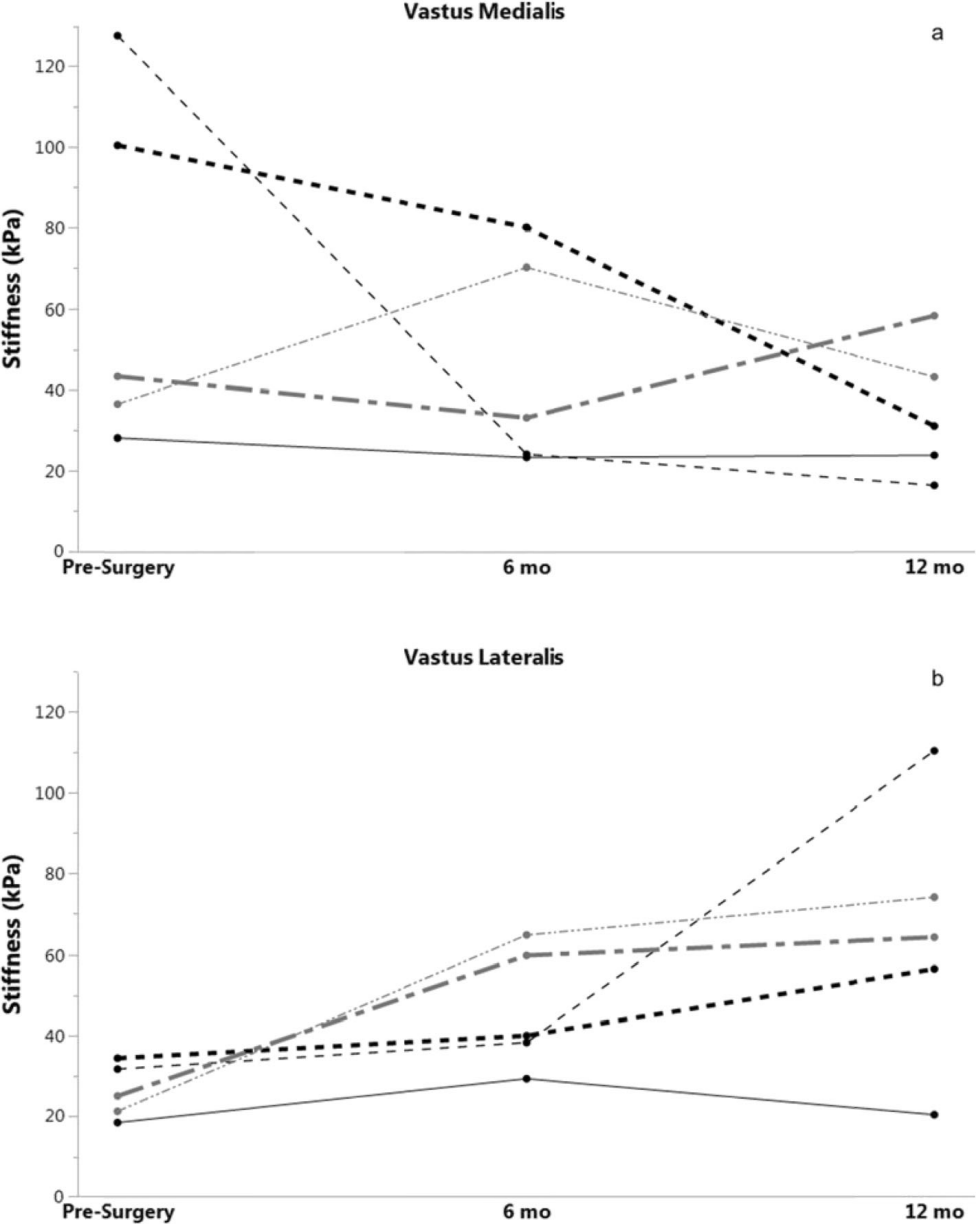
Longitudinal changes of quadriceps musculature stiffness in ACL-injured subjects for a) Vastus Medialis and b) Vastus Lateralis. Each line represents a unique subject. 10% MVIC trial is shown. kPa, kilopascal

No meaningful differences were observed between stiffness and average rate coding. Average rate coding appears clustered within a similar range for all groups; however, stiffness exhibited much greater variability in range, even within the same group, than average rate coding. Due to poor subject follow-up and data loss, conclusions about longitudinal changes after ACLR could not be statistically tested since only five subjects were available with complete data. However, changes in stiffness within individuals longitudinally after ACL injury were explored. Across time after ACLR, active VL stiffness appeared to generally increase whereas VM stiffness tended to decrease (Fig. 6). It is interesting to note that the relationship between MU rate coding and stiffness appears more disorganized and variable for the quadriceps than the hamstrings. Clinical significance, such as the effect on strength recovery or the risk for second ACL injury, remains to be determined for the relationship between stiffness and MU activity. EMG decomposition at low levels of force is limited due to lower signal-to-noise ratio; future testing with improved SWE capabilities may better establish a relationship between stiffness and MU rate coding after injury.

Several studies have evaluated biomechanical and neuromechanical outcomes (as measured by SWE and/or EMG decomposition) after upper extremity injuries or in neuromuscular disorders [12], but to the authors’ knowledge, not after ACLR. All but one study reported either an increase or decrease in stiffness relative to controls; no differences were reported in subjects with self-reported symptoms of neck and shoulder stiffness [12, 33]. However, the diagnostic utility of SWE in ACL-injured patients remains limited due to the large range of stiffness values that can be generated during active contraction and the limited range of SWE capability. SWE performance at higher levels of force and the resultant higher stiffness was unreliable and therefore could not be used to better delineate relationships at higher force levels. This is also observed in the large spread of the SWE data, even at lower %MVIC trials. Improvement in SWE capabilities will improve data quality and reduce variability attributed to measurement errors. Recent development of a shear wave tensiometer to assess muscle-tendon loads may provide an alternative solution to the SWE limitations discussed [34]. While the current methodologies restricted assessment of stiffness and MU activity to isometric tasks, athletic activities are dynamic movements and pre-defined force levels are not achieved. These technologies and results give insight into how the athlete may perform, but cannot be extrapolated to a dynamic athletic task or environment. Improved methodology capabilities and data quality will allow for a better understanding of the variability between muscles, trials, and subject groups, particularly in a dynamic athletic environment.

## Conclusion

In conclusion, thigh musculature stiffness changed throughout rehabilitation and remained altered at 12 months after ACLR. With improved SWE and EMG decomposition technology, these methodologies may be incorporated into future clinical examination and research to evaluate the effectiveness of rehabilitation after ACLR to restore neural activity and stiffness.

## Data Availability

The corresponding author has full access to all data in the study and assumes final responsibility for the publication. The dataset used and analyzed during the current study, which are maintained by designated researchers, are available through the corresponding author upon reasonable request.

## Abbreviations

ACL: Anterior cruciate ligament
ACLR: Anterior cruciate ligament reconstruction
BF: Biceps femoris
EMG: Electromyography
F_max_: Maximum strength
kPa: Kilopascal
MU: Motor unit
MVIC: Maximal voluntary isometric contraction
SWE: Shear wave elastography
ST: Semitendinosus
VL: Vastus lateralis
VM: Vastus medialis
